# The effect of coronal splits on the structural stability of bi-condylar tibial plateau fractures: a biomechanical investigation

**DOI:** 10.1007/s00402-020-03412-8

**Published:** 2020-03-26

**Authors:** Shabnam Samsami, Robert Pätzold, Martin Winkler, Sven Herrmann, Peter Augat

**Affiliations:** 1 Laboratory of Biomechanics and Experimental Orthopaedics, Department of Orthopedic Surgery, Physical Medicine and Rehabilitation, University Hospital of Munich (LMU), Campus Grosshadern, Munich, Germany; 2 Institute for Biomechanics, Berufsgenossenschaftliche Unfallklinik, Murnau, Germany; 3 Department of Trauma Surgery, Berufsgenossenschaftliche Unfallklinik, Murnau, Germany; 4 Institute for Biomechanics, Paracelsus Medical University, Salzburg, Austria

**Keywords:** Bi-condylar tibial plateau fracture, Coronal fracture line, Horwitz fracture model, Coronal fracture model, Mechanical test, Interfragmentray displacement

## Abstract

**Introduction:**

Surgical treatment of bi-condylar tibial plateau fractures is still challenging due to the complexity of the fracture and the difficult surgical approach. Coronal fracture lines are associated with a high risk of fixation failure. However, previous biomechanical studies and fracture classifications have disregarded coronal fracture lines.

**Materials and methods:**

This study aimed to develop a clinically relevant fracture model (Fracture C) and compare its mechanical behavior with the traditional Horwitz model (Fracture H). Twelve samples of fourth-generation tibia Sawbones were utilized to realize two fracture models with (Fracture C) or without (Fracture H) a coronal fracture line and both fixed with lateral locking plates. Loading of the tibial plateau was introduced through artificial femur condyles to cyclically load the fracture constructs until failure. Stiffness, fracture gap movements, failure loads as well as relative displacements and rotations of fracture fragments were measured.

**Results:**

The presence of a coronal fracture line reduced fracture construct stiffness by 43% (*p* = 0.013) and decreased the failure load by 38% from 593 ± 159 to 368 ± 63 N (*p* = 0.016). Largest displacements were observed at the medial aspect between the tibial plateau and the tibial shaft in the longitudinal direction. Again, the presence of the coronal fracture line reduced the stability of the fragments and created increased joint incongruities.

**Conclusions:**

Coronal articular fracture lines substantially affect the mechanical response of tibia implant structures specifically on the medial side. With this in mind, utilizing a clinically relevant fracture model for biomechanical evaluations regarding bi-condylar tibial plateau fractures is strongly recommended.

## Introduction

Bi-condylar tibial plateau fractures are challenging traumas due to their complex fracture geometry and accompanying soft tissue injury [1, 2]. These fractures mainly occur in young patients as a result of high-energy trauma and generally require open reduction and internal fixation [3, 4]. Healing complications for these fractures have been reported from 14% up to a staggering 42% [5–10]. The main goal of operative treatment is the patient’s return to daily activity and functionality, which can be achieved through accurate reconstruction of the knee joint and the anatomical axes [2]. For planning and achieving suitable treatment, it has been recognized that providing a three-dimensional representation of the fracture by CT imaging plays a critical role [1, 11–14]. This is particularly important for articular fractures in the coronal plane as they are difficult to detect on bi-planar radiographs and complicated to characterize by two-dimensional fracture classifications such as Schatzker or AO/OTA [15]. The AO/OTA and Schatzker classifications are the most common taxonomies of tibial plateau fractures due to their simplicity, while they disregard injury patterns observed in the third dimension [16].

The clinical relevance of these coronal fractures was identified by Barei et al. as a fracture pattern that separates a posteromedial fragment from the tibial plateau [11] and which has a prevalence of almost 50% in complex tibial plateau fractures [1, 11, 17]. The detection of this fracture line is clinically important because lateral locking plates, which are a common fixation method for this fracture, may not effectively stabilize the posteromedial fragment and supplemental implants may be required [5, 6, 11, 18–20]. To adequately describe the personality of complex tibia plateau fractures, various three-dimensional classification schemes such as the “three-column” concept were developed to refine the traditional planar classifications as a guide for surgical planning [21]. Recently, an extension of the Schatzker classification was introduced in which the fracture type and the mechanism of injury were described based on plain radiographs as well as CT data were utilized to provide complemental third-dimensional information about the location of the main fracture planes [22]. Although the clinical relevance of the posteromedial fragment and the dependency of treatment plans on identifying fracture locations has been recognized, there is still a lack of understanding regarding the biomechanical implications of the posteromedial fragment, in particular with respect to its adequate stabilization [1]. Previous biomechanical studies on bi-condylar tibial plateau fractures have largely been based on the model developed by Horwitz et al. [23] to simulate a Schatzker Type VI fracture. As this model is based on a coronal projection of the fracture, it completely ignores any coronal fracture lines and thus the presence of a posteromedial fragment. Yet, it remains the most frequently employed biomechanical model [23–35] on which recommendations for fracture fixation of complex tibia plateau fractures are based upon.

Due to the aforementioned negligence of articular fracture lines, establishing a clinically relevant, biomechanical fracture model is required to resolve controversies regarding the ideal fixation method for this complex trauma. The aim of this study was to develop a biomechanical model for bi-condylar tibial plateau fractures, which incorporates a coronal fracture line. We hypothesized that our novel coronal fracture model would exhibit inferior mechanical stability compared to the traditional Horwitz model.

## Materials and methods

This biomechanical study was performed on synthetic bone analogues which were osteotmized to produce two different fracture models: the traditional Horwitz fracture model (Fracture H) and a novel fracture which was based on a systematic review of CT images [1] and included a coronal fracture line (Fracture C). The fracture models were fixed with locked plating constructs and were quasi-statically as well as cyclically loaded to determine the mechanical stability as measured by stiffness, fragment movement and failure loads.

### Sample preparation

**Fig. 1:**
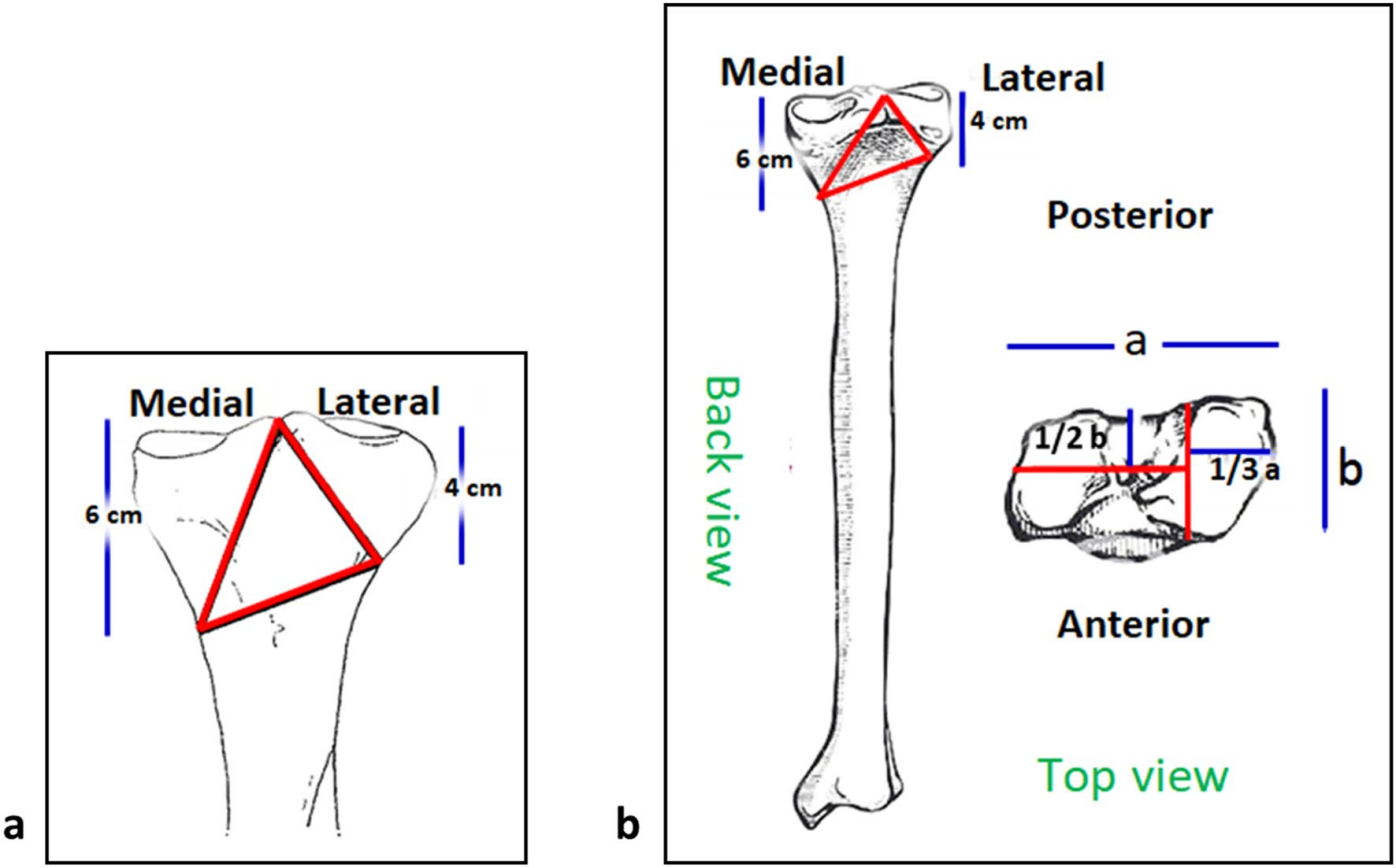
**a** Fracture H according to the schematic drawing of Horwitz et al. [23] and [34]. **b** Fracture C developed based on clinically relevant fracture lines [1]

Twelve synthetic tibial bones (#3406 left large tibia, 4th Generation, Sawbones, Malmö, Sweden) were prepared with identical osteotomies using a custom-made jig and an oscillating saw. For Fracture H, the central triangle of the proximal tibia was removed to mimic an unstable fracture situation [23, 34]. The first cutting line started from the intercondylar eminence and ended at a point on the lateral cortex located 4 cm distally from the lateral plateau. The medial cut was made from the intercondylar eminence to a point on the medial cortex positioned 6 cm distally from the medial plateau. A final cut was made to connect the lateral and medial cortex points (Fig. 1a). The coronal fracture model (Fracture C) consisted of coronal and sagittal articular fracture lines. The coronal fracture line was made in the center part of the medial tibial plateau in the superior view. The sagittal fracture line split the lateral plateau and intercondylar eminence of the tibia in the transverse plane. Then, lateral and medial cuts were made from the Tuberculum intercondylare laterale to the lateral cortex at 4 cm and on the medial cortex at 6 cm distal from the tibial plateau. The final osteotomy connected the lateral and medial splits and the central triangle of the bone from the proximal tibia was removed (Fig. 1b).

**Fig. 2:**
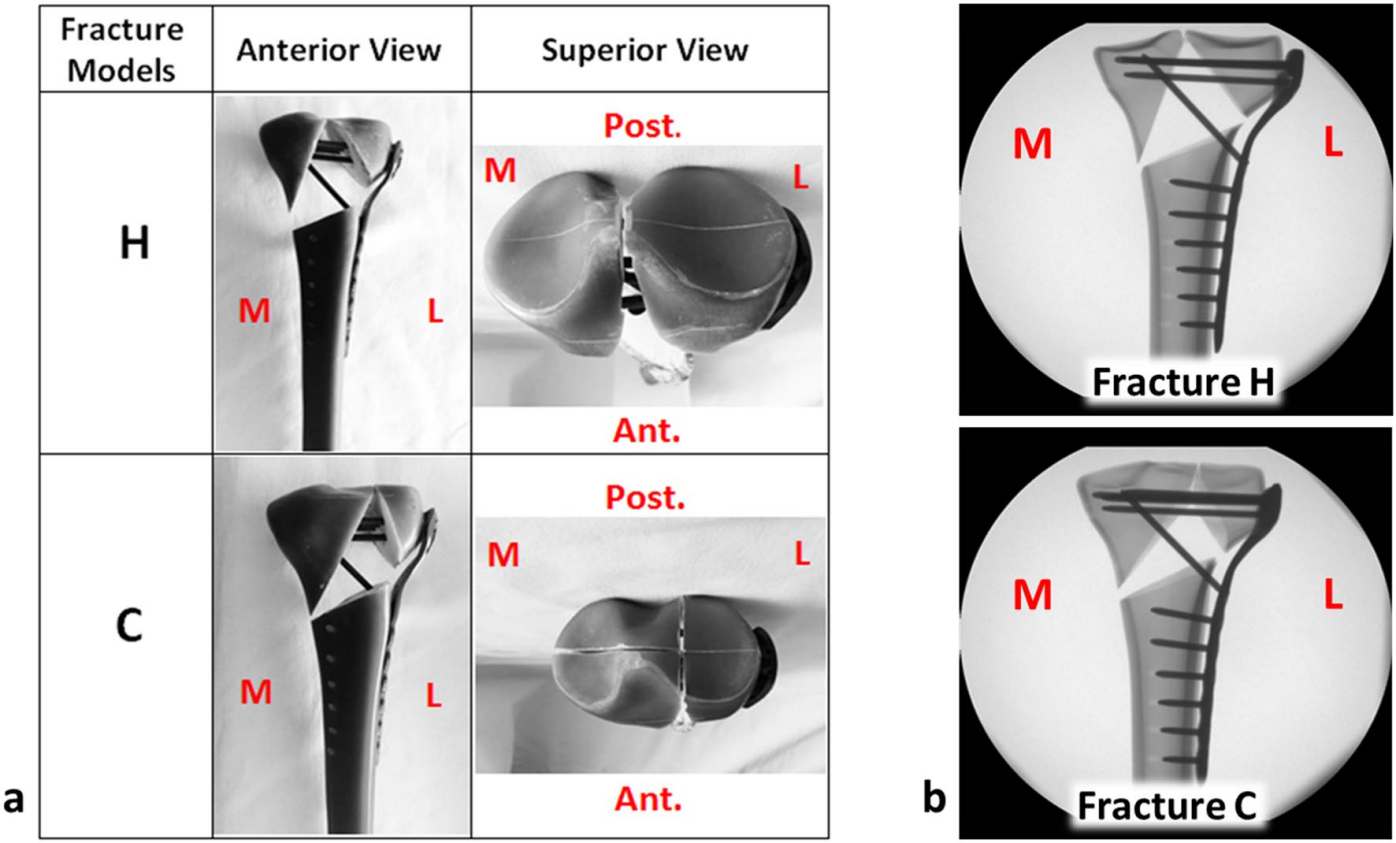
**a** Prepared samples for fracture models H and C. **b** Anterior–posterior X-ray of prepared specimens for each fracture model

Both fracture types were fixed with titanium locking plates (AxSOS Proximal Lateral Tibia Plate, left, six-hole length, Stryker, Selzach, Switzerland) by an experienced orthopedic trauma surgeon according to the manufacturers’ recommendations using locking self-taping screws of 4-mm diameter (four articular screws including two 80-mm screws for proximal-posterior and proximal-inferior plate holes, 85 and 70-mm screws for proximal-middle and proximal-anterior plate holes, respectively, one kick-stand screw with 75-mm length as well as six shaft screws with length between 20 and 32 mm). A 3D-printed template was used to ensure that implants were inserted identically in all specimens (Fig. 2).

### Experimental setup

**Fig. 3:**
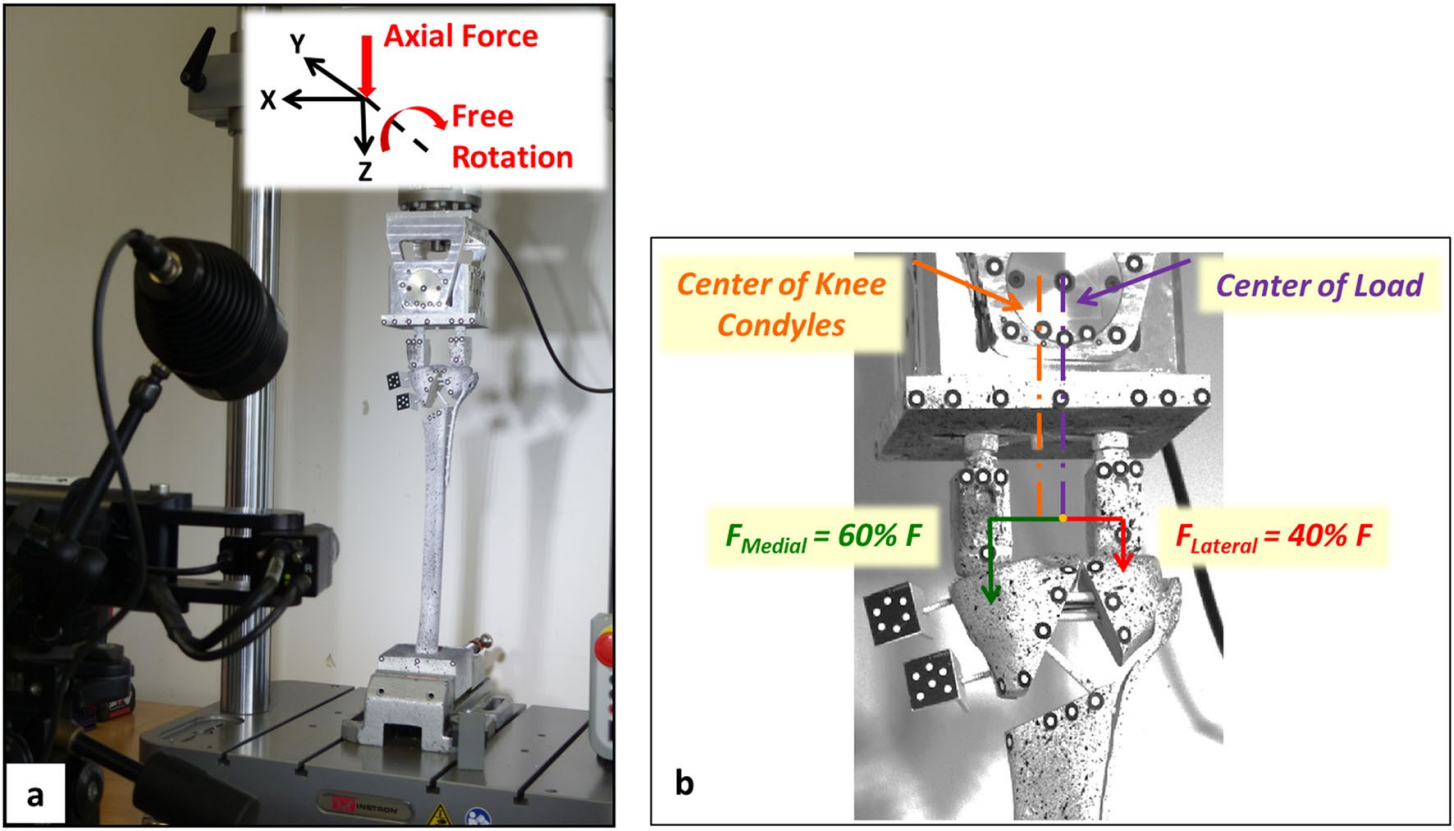
**a** Experimental setup for mechanical test in which the highlighted local coordinate system indicates degrees of freedom for the load applicator. **b** Load applicator and more details about load distribution. Also, the marker placements and the spray pattern for image detection can be seen

The distal end of the tibia was embedded in an aluminum box to a depth of 50 mm using a three-component casting resin (RenCast FC 53 A/B + Füller DT 082, Gößl + Pfaff GmbH, Karlskron/Brautlach, Germany). This box was clamped to the base of the testing machine (Instron E3000, Instron Structural Testing, High Wycombe, UK) to rigidly fix samples in a vertical position. Loading was introduced through artificial femur condyles of unilateral knee replacements which were embedded in polyurethane blocks. These blocks were attached to the actuator of the testing machine with a hinge joint, which allowed the femur condyles to tilt in the frontal plane and balance the movement of the tibial head (Fig. 3a). The testing machine included a Dynacell load cell (Capacity of ± 10 kN, ISO 7500-1 Class 1, Instron Structural Testing, High Wycombe, UK) and data logging software (Instron Console V8.4 and Instron Wave Matrix V1.5, High Wycombe, UK).

To simulate physiological loading conditions, femoral condyles were positioned in such a way that the equidistant point and the axis of the testing machine were not aligned, so the applied load was distributed 40% laterally and 60% medially on the tibial plateau (Fig. 3b) [35, 36].

### Loading scenario

**Fig. 4:**
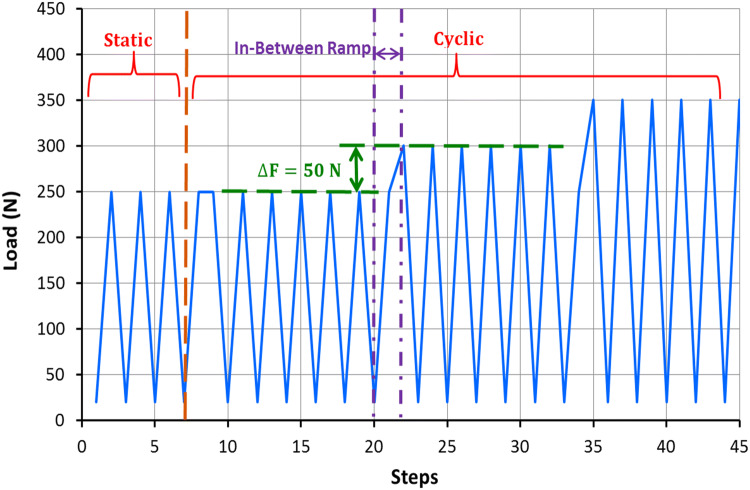
Force-steps curve that indicates loading scenario includes static and incremental cyclic

At the beginning of loading, six static displacement-controlled ramps (10 mm/min) up to 250 N were performed. The first three static ramps allowed the samples to settle, while the second three cycles were used to measure the initial stiffness of the tibia-implant constructs. Afterwards, constructs were cyclically loaded with a sinusoidal axial load (2 Hz) between the lower level of 20 N and an incrementally increasing upper load level. The upper load level started at 250 N and was increased stepwise by 50 N every 500 cycles to mimic increasing levels of weight bearing. Additionally, static measurements were taken at maximum loads before and after increments of 500 cycles (Fig. 4).

All specimens were loaded until mechanical failure (i.e., Sawbone breakage, gap closure on the medial side of the tibia, or implant failure). With a retrospective analysis of ARAMIS data, the clinical failure point was defined as ≥ 5° relative rotations of fracture fragments, ≥ 5-mm fracture gap displacements for medial- or lateral-shaft gaps, or ≥ 2-mm displacements of articular gaps on the tibial plateau, whichever occurred first [37, 38].

### Interfragmentary movement analysis

**Fig. 5:**
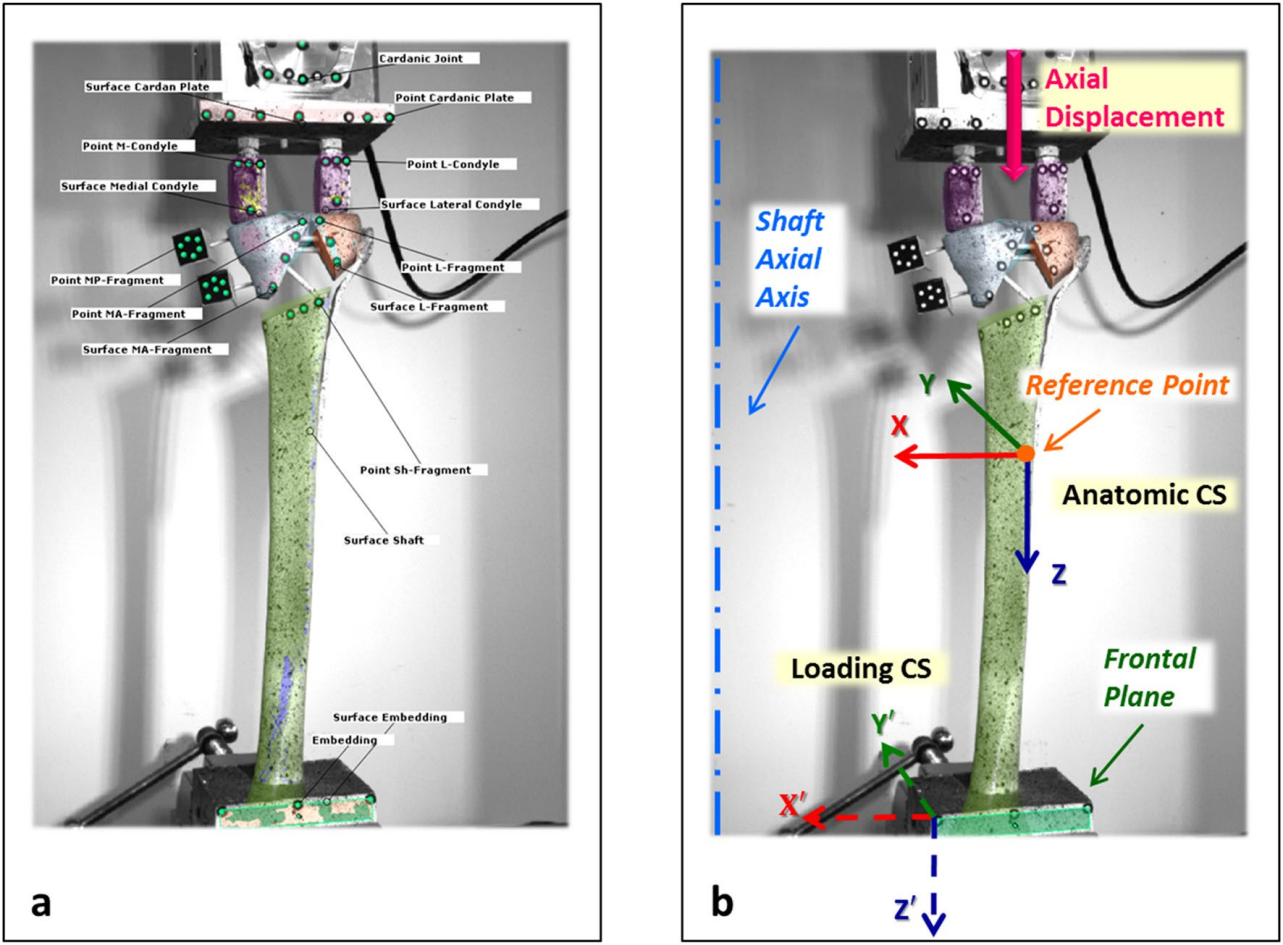
**a** A general view for the defined markers and surfaces as well as fitted CAD files in GOM Correlate software. **b** Axial displacement of the loading point highlighted with an arrow as well as details of the loading and anatomic coordinate systems

To track the relative displacement of fracture fragments, an optical measurement system (ARAMIS 5M, GOM GmbH, Braunschweig, Germany) with measurement error < 0.1% and 2% for relative translational and rotational movements, respectively [39], was utilized. ARAMIS system consists of data capturing and analysis software (GOM Correlate Professional 2018, GOM GmbH, Braunschweig, Germany) that utilizes stereo-image based techniques to evaluate the coordinates and displacements of objects with image correlation from point marker or stochastic pattern recognition. The global coordinate system was defined using computer-aided design (CAD) files of the tibial shaft and the best-fit algorithm included in the GOM Correlate software. Then, CAD files of other fracture fragments were virtually matched with the corresponding surface and point components to track relative displacements between fracture parts (Fig. 5a).

**Fig. 6:**
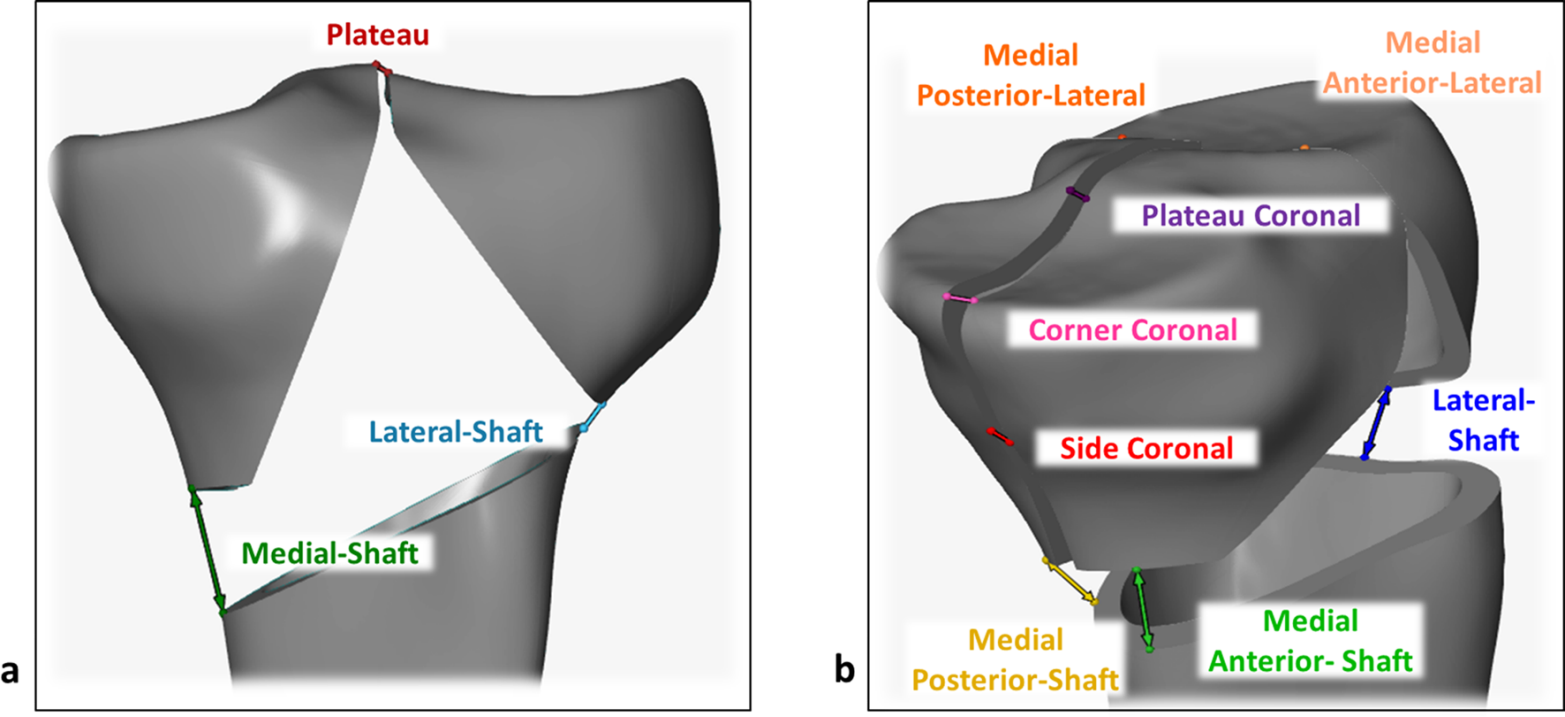
Positions for measurement of interfragmentary movements. **a** Fracture H: medial-shaft, lateral-shaft, and plateau gaps. **b** Fracture C: medial anterior-shaft, medial posterior-shaft, lateral-shaft, side coronal, corner coronal, plateau coronal, medial anterior-lateral, and medial posterior-lateral gaps

Interfragmentary movements and the relative rotation of fracture segments with respect to the tibial shaft were analyzed to elucidate displacement of fracture fragments. All kinematic data were reported in the anatomic coordinate system in which *x*, *y* and *z* axes indicate frontal, sagittal and longitudinal directions, respectively. Moreover, the axial displacement of the loading center was evaluated in the loading coordinate system (Fig. 5b). Every 100 cycles, ARAMIS pictures were taken at the maximum and minimum loads to measure the elastic and plastic deformations of the construct, respectively. Additionally, a static measurement at the point of maximum load before and after each 500 cycles was made to track the elastic deformation of the specimens. To evaluate the movement of the fracture fragments during loading, a pair of points was considered in the center of each gap to measure changes in the relative distances of individual gaps (Fig. 6).

### Measurement outputs

From recorded data, the following parameters were evaluated:

1. Static construct stiffness (N/mm), defined as load changes between 20 and 250 N divided by the corresponding axial displacement of the loading center in the static step.
2. Cyclic construct stiffness (N/mm), which was calculated by considering in-between ramps between cyclic steps and dividing load changes between 20 and 250 N by the corresponding axial displacement of the loading center.
3. Interfragmentary displacement (mm), determined as relative movement between individual fracture gaps and reported for static loading as well as after 2500 cycles at the 500 N force corresponding to 20% of maximum knee contact force [40].
4. Rotations of fracture fragments around the three anatomical axes with respect to the tibial shaft (degree), which were evaluated at the maximum load levels during cyclic steps.
5. Failure load (N) and failure cycles that determine the load and the number of cycles where the samples exceeded clinical failure criteria.
6. Survival curves as a comparison between fracture models for level of load tolerance regarding clinical failure.

### Statistical analysis

Independent *t* tests were applied to compare mechanical parameters between the two fracture models. The assumptions of independent *t* test consisting of independence, interval scale, normal distribution, as well as homogeneity of variances (Levene’s test) were evaluated for both fracture groups. Normal distributions of data were assessed with Shapiro–Wilk’s test (*p* > 0.05) as well as visual inspection of the histogram, normal *Q*–*Q*, and box plots. For the fatigue tests, Kaplan–Meier survival analyses with log-rank tests were executed (IBM SPSS Statistics 19, Chicago, IL, US).

## Results

**Fig. 7:**
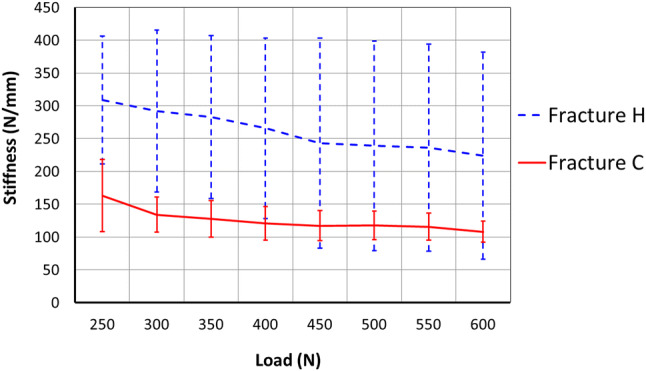
Cyclic stiffness for both fracture configurations (mean ± standard deviation, *n* = 6)

In static loading, the axial stiffness of Fracture C was 43% lower than that of Fracture H (Table 1, *p* = 0.013). During cyclic steps, depending on the load level, Fracture C was on average 47–55% laxer than Fracture H (Fig. 7).

**Table 1:** Comparing mechanical properties between Fractures H and C (mean ± standard deviation, *n* = 6)

	Static stiffness (N/mm)	Failure load (N)	Failure cycles
Fracture H	304 ± 95	593 ± 159	3517 ± 1493
Fracture C	172 ± 50	368 ± 63	1433 ± 656
*p*	0.013	0.016	0.017

**Fig. 8:**
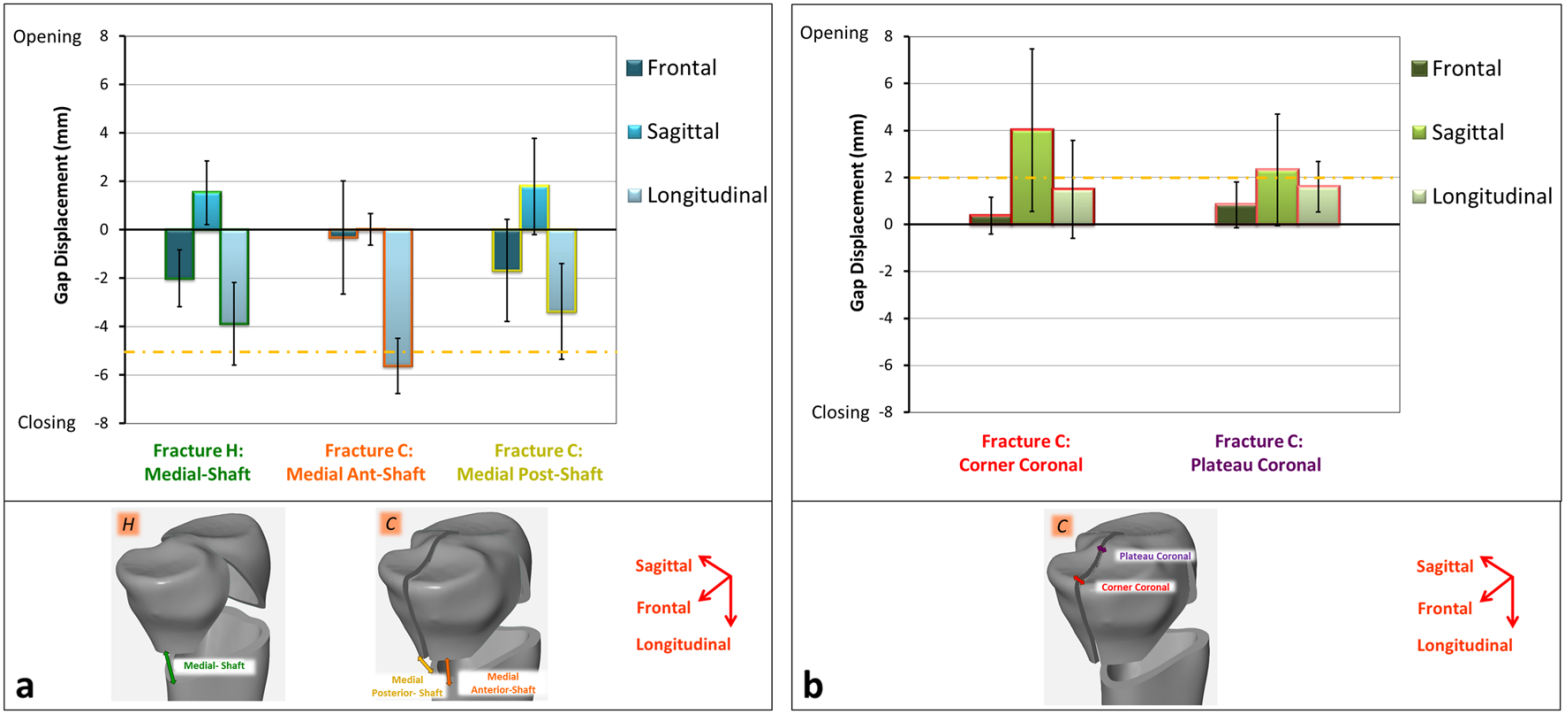
Interfragmentary displacements at 500-N cyclic load after 2500 cycles. **a** Medial-shaft gaps of Fractures H and C. **b** Coronal gaps of Fracture C. The dash-dotted lines depict clinical failure criteria

To elucidate the effect of the coronal fracture line on fragment stability, relative displacements and rotations were evaluated for the key fracture fragments. Although fragment displacements were evaluated during the whole cyclic testing procedure, we report the displacements after 2500 cycles at the 500-N load level corresponding to 20% of the maximum knee contact force (Fig. 8). For both Fractures H and C, displacements on the medial sites were predominantly in the longitudinal direction followed by frontal and sagittal displacements. In particular, displacement at the medial anterior-shaft gap of Fracture C was almost exclusively in longitudinal direction and exceeded the 5-mm clinical failure criteria (Fig. 8a). Both coronal gaps of Fracture C were mainly displaced in the sagittal direction followed by the longitudinal and frontal movements. The average sagittal displacements exceeded the 2-mm failure criteria at both locations in the plateau (Fig. 8b).

**Fig. 9:**
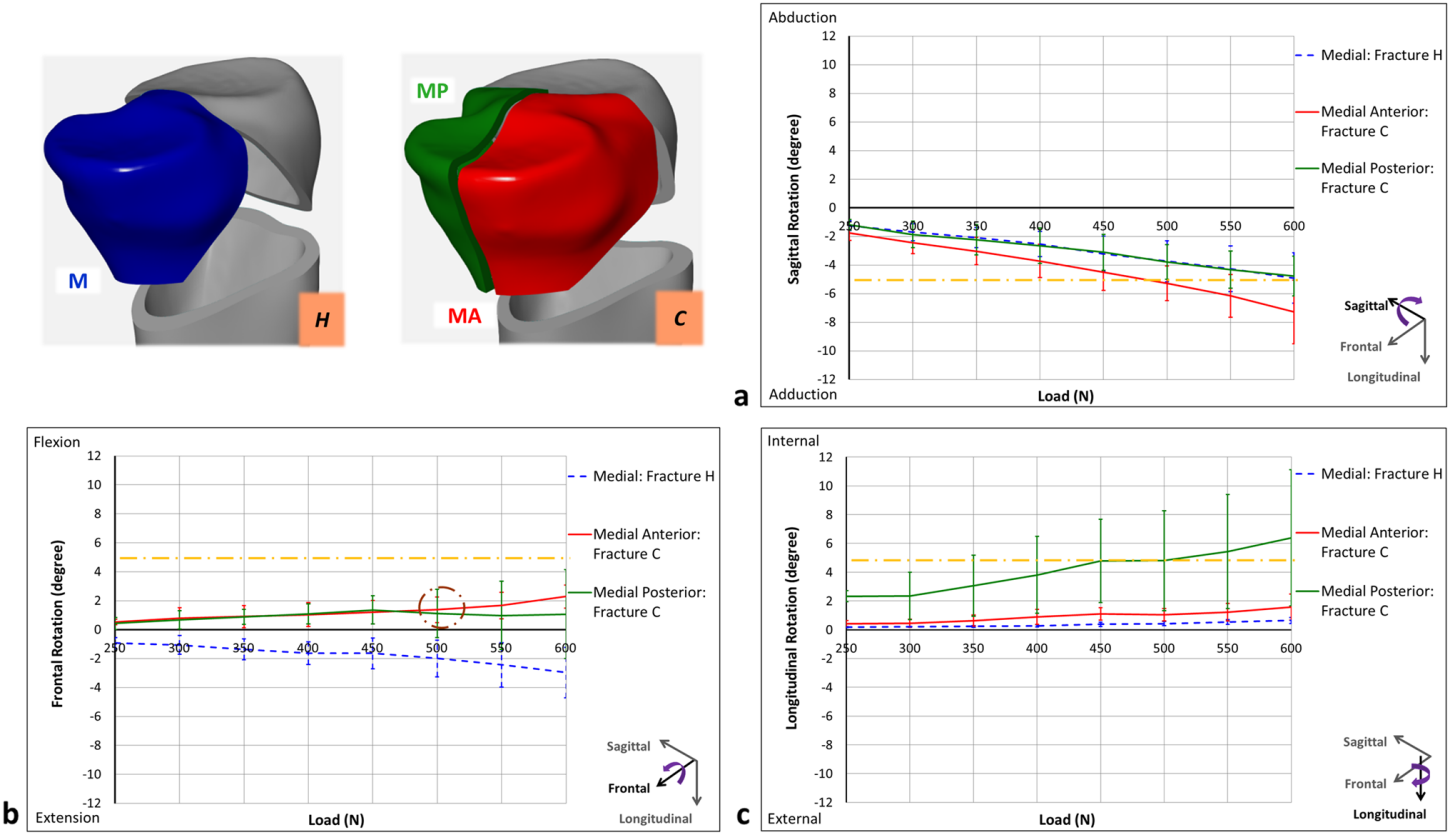
Relative rotations of the medial fragments for Fractures H and C with respect to the tibial shaft. **a** Sagittal rotation. **b** Frontal rotation (dash-dotted circle indicates destabilization of diagonal screw). **c** Longitudinal rotation. Dash-dotted lines demonstrate clinical failure criteria

As the displacement measurements indicated that the medial-anterior and medial-posterior fragments of Fracture C moved in different directions, relative rotations of these fragments were analyzed (Fig. 9). Analysis of the sagittal rotation revealed that the coronal split mainly destabilized the medial-anterior fragment which showed larger rotations compared to its corresponding posterior one as well as the whole medial segment of Fracture H (Fig. 9a). Regarding the frontal rotation, unlike the movement of the medial segment in Fracture H, the coronal split resulted in reversing the rotation of both medial fragments toward the posterior direction (Fig. 9b). Finally, for the longitudinal rotation, the coronal split destabilized the medial-posterior fragment, demonstrating a more than threefold increase in internal rotation when compared to its anterior counterpart as well as the medial component of Fracture H (Fig. 9c). For both Fractures H and C, interfragmentary movements of the lateral-shaft and medial–lateral gaps were almost negligible compared to those of the medial-shaft and coronal gaps. Moreover, the movements of these two gaps and relative rotations of lateral fragments were similar in both fracture models.

**Fig. 10:**
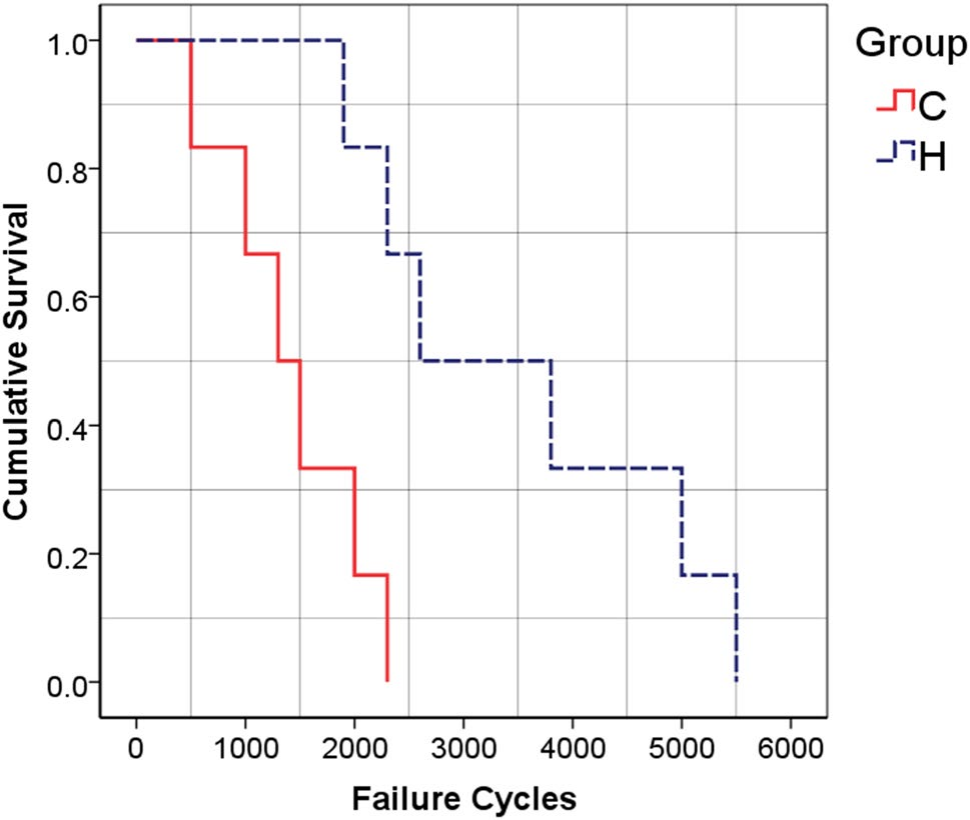
The survival curves for Fractures H and C

Shapiro–Wilk’s test (*p* > 0.05) as well as visual inspection of the histogram, normal *Q*–*Q*, and box plots indicated that for both Fractures H and C, the parameters of mechanical stability (i.e., static stiffness, failure load, and failure cycles) were approximately normally distributed. According to the clinical failure criteria, Fracture C failed earlier and at lower load levels compared to Fracture H which tolerated 60% higher load levels (*p* = 0.016). Failure cycles of Fracture H samples were almost 2.5 times that of Fracture C (Table 1, *p* = 0.017). The survival analysis revealed that the survival rate of Fracture H was almost 2.5 times that of the Fracture C group. While none of the Fracture C samples survived the clinical failure criteria until 2500 cycles or 500-N load, half of Fracture H specimens survived 2500 cycles (Fig. 10, *p* = 0.006).

## Discussion

Articular fracture lines in complex tibial plateau fractures which result in a coronal split have been widely ignored in previous biomechanical studies and fracture classifications [11]. Thus, we developed a coronal fracture model based on 3D fracture morphologies [1] and assessed this model biomechanically. Our study revealed that the presence of a coronal split generates an unstable posteromedial fragment which is inadequately stabilized with unilateral plating from the lateral aspect. The coronal split reduced the stiffness and decreased the strength of fracture fixation and resulted in increased fragment displacements during loading.

The biomechanical assessments revealed that Fracture H was almost two times stiffer and tolerated 1.5 and 2.5 times higher load level and cycles, respectively, as compared to Fracture C (Table 1). Also, the survival rate of the Fracture H group was nearly 150% higher than that of Fracture C. Thus, the coronal fracture line remarkably destabilized the fracture fixation construct.

To further understand the effects of coronal fracture lines, the rotations of fracture fragments relative to the tibial shaft and interfragmentary displacements were analyzed. Interfragmentary movements (especially on the articular surfaces) are considered an important clinical parameter because an articular subsidence of more than 2–3 mm remarkably increases knee joint pressure and may result in osteoarthritis [41, 42]. Moreover, malalignment is an important factor in evaluating treatment outcomes of tibia plateau fractures and rotations of joint lines more than 5° are considered fixation failure [37, 43, 44]. Thus, interfragmentary displacements of the medial and plateau fracture gaps were analyzed to assess a risk of bone non-union and osteoarthrosis, respectively. The relative rotations of medial fracture fragments with respect to the tibial shaft are also indicative of limb malalignment. To the best of our knowledge, this was the first study in which 3D displacements of fracture fragments have been evaluated for complex tibial plateau fractures. To build upon previous investigations regarding bi-condylar tibial plateau fractures, which only evaluated the facture fragment subsidences [23–31, 33, 45] or the frontal intra-articular gap movements [35], we presented a comprehensive 3D kinematic evaluation of all fracture fragments to illuminate the effects of coronal fracture lines.

During cyclic loading, the medial-anterior segment of Fracture C mainly moved in the longitudinal direction and exceeded 5-mm clinical failure which is indicative of a high risk for bone non-union. At the same time, the medial-posterior portion separated from the anterior one in the posterior direction with sagittal displacements exceeding 2 mm which indicates a risk of knee osteoarthritis (Fig. 8).

Due to the vertical nature of the coronal fracture line, the medial segments of Fracture C were subjected to higher shear forces [11]. Therefore, compared to Fracture H, the medial segments of Fracture C, especially medial-posterior one, demonstrated higher instability (Fig. 9). From sagittal rotational disparity between the two medial fragments, it can be concluded that coronal fracture line results in an articular step-off which may lead to knee osteoarthritis. Since sagittal rotations occurred due to screw bending, the coronal split mainly destabilized the anterior fragment perhaps due to the inclusion of fewer screws in comparison to its posterior counterpart. Regarding the frontal rotations, the contact between the load applicator and the coronal edges of the medial fragments in Fracture C resulted in reversing the movement of these fragments compared to Fracture H. For Fracture C in the 500-N cyclic load level, the diagonal screw which is the only connection between the medial fragments and mainly passes through the medial-posterior segment, lost its connection with the anterior one. Thus, medial-anterior fragment rotated in frontal direction, while the medial-posterior part remained stable. Importantly, longitudinal rotations demonstrate that the medial-posterior and medial-anterior fragments of Fracture C tilted away towards the posterior direction of the transverse plane. This separation resulted in higher loading on the medial-posterior part and consequently more longitudinal rotation compared to the medial-anterior one. It should be noted that for Fracture C there is a risk of malalignment in frontal and transverse planes due to exceeding the clinical failure criteria for sagittal and longitudinal rotations of the medial-anterior and medial-posterior fragments, respectively.

These biomechanical findings demonstrated that disregarding the coronal fracture line will result in overestimation of structural rigidity of tibial plateau fracture constructs. Also, the relative displacement of fracture fragments was considerably affected by the presence of a coronal split. While the medial fragment of Fracture H displaced mainly in the frontal plane, the coronal fracture resulted in rotational instabilities of both medial fragments. Therefore, with the presence of the coronal split, there is a risk of non-union at the tibial medial side as well as malalignment of the tibial plateau and eventually osteoarthrosis. With kinematic evaluations in mind, these consequences are likely to occur at lower load levels in Fracture C than in Fracture H. Noticeably due to higher relative rotations for the medial-posterior fragment and interfragmentary movements over 5 mm for medial anterior-shaft gap, an additional medial fixation could provide higher stability for Fracture C. These conclusions agree with previous clinical studies that have demonstrated higher clinical failure rates at the medial side of complex tibial plateau fractures [1, 2]. Also, it has been clinically [2, 11, 17, 19, 20] or biomechanically [45] observed that lateral locking implants may not adequately stabilize the posteromedial fragments of bi-condyla tibia plateau fractures and a supplementary medial implantation will be required.

Considering the clinically observed importance of posteromedial fragment for bi-condylar tibia plateau fractures, developing a biomechanical fracture model including the coronal fracture line is highly demanded [11]. Contrary to previous experimental studies, which applied loads only on the medial tibial plateau [23, 29, 32–34, 45], our load applicator was designed to simultaneously apply axial forces on both tibial plateau surfaces and distribute it 40% laterally and 60% medially, corresponding to physiological conditions [35, 36]. This load applicator with the adjustable position of the medial and lateral indenters can be used even for cadaveric samples with various plateau widths. Moreover, using artificial femur condyles provided a more physiological contact pressure on the tibial plateau as compared to previous biomechanical studies [35]. Furthermore, like some of previous investigations [23, 26, 31], the fracture model of this study presented a highly unstable situation by removing the central triangle of bone from the proximal tibia. Comparing mechanical behaviors of the Horowitz fracture model among previous experimental studies [23, 26, 29, 31, 33, 35] is difficult due to some distinctions regarding sample types (composite or cadaveric bones), loading and boundary conditions as well as loading scenarios. In this regard, only the study of Lasanianos et al. [35] was almost similar to ours. The static stiffness of Fracture H was found to be 304 $\pm$ 95 N/mm which is in the same range of their reported value (400 $\pm$ 64 N/mm) [35]. The mentioned research group obtained higher static stiffness values for specimens fixed with lateral locking plates. A reason for this could be that the fracture model of their study was stiffer due to preserving the middle fracture gap. Also, a different distal boundary condition was assumed in their experimental setup. Failure load of Lasanianos et al.’s fracture model was almost three times that of Fracture H reported in the current research, since we assumed the clinical failure criteria which is in contrast with their mechanical failure criteria. Among previous biomechanical studies, only the investigation of Yoo et al. [45] considered a Horwitz fracture model consisting of the posteromedial fragment. However, their fracture model was different than our coronal fracture model developed based on the clinical review of Pätzold et al.’s study [1]. Fracture C includes a sagittal articular fracture line in addition to the proximal fracture gap that is located laterally compared to that of Yoo et al.’s fracture model. They reported failure load of the Sawbone samples fixed with the tibial less invasive stabilization system (LISS) to be 1680 ± 179 N that is almost 4.5 times that of Fracture C. This difference could be due to their stiff fracture model in which the middle fracture gap was preserved as well as the lateral articular split was not included. Additionally, they utilized a hemispherical impactor for exclusive loading on the medial tibial plateau, although our loading was performed with a dual applicator which simultaneously applies the axial force on both tibial plateaus. Moreover, their failure criteria were different than our clinical ones. Regarding the loading scenario, an incremental cyclic loading until failure was considered in this study. Unlike previous studies, in which the testing protocol did not include the incremental cyclic loading [23–35], the maximum load level of our gradual fatigue test increased stepwise by 50 N every 500 cycles. We believe that this progressive cyclic loading can simulate daily living activities of patients during the healing process, since after surgery incremental weight bearing on the injured limb is recommended.

Naturally, this study had some limitations which should be taken into consideration. First, artificial fourth-generation Sawbone samples were used for mechanical testing. Due to their material properties, their failure behavior might potentially differ from failure in human bone specimens. That is why we focused the failure analysis on relative movements between fragments rather than catastrophic failure of the fracture fixation construct. Also, Sawbone provided consistency in material, geometry, and mechanical properties, which increases the power to detect differences between groups. Second, the two fracture models were compared only under axial and bending loading conditions. The load applicator used in this study provided the sagittal rotational degree of freedom and consisted of femoral components of unilateral knee joint replacements which applied physiological pressure to the tibial plateau surface with distribution of 60% on the medial side and 40% laterally. In addition, the loading scenario only included axial knee joint forces, although for more realistic loading, the effect of muscle forces should also be considered. Moreover, instead of simulating the ankle joint, specimens were fixed distally to the testing machine directly, resulting in a somewhat overconstrained loading condition. Furthermore, to evaluate displacements and rotations, the fracture fragments were assumed as rigid parts. This assumption could be used due to high stiffness of the Sawbones which makes local deformations of segments negligible. Additionally, we simulated a hypothetical, unstable bi-condylar tibial plateau fracture in both fracture models by removing the central triangle of the proximal tibia as well as the side gap from the medial tibia. Last but not least, only one particular implant configuration with single lateral plating has been considered. With a more stable configuration like double medial and lateral platting which stabilizes the posterior fragment, the destabilizing effect of the coronal fracture is most likely less pronounced.

## Conclusion

The outcomes of this study emphasize that it is mandatory for biomechanical simulations regarding complex tibial plateau fractures to be based on a clinically relevant fracture model such as ours (Fracture C) due to its ability to mimic native mechanical behavior more accurately than the traditional Horowitz model. The observed instability on the medial side of the coronal fracture model suggests that lateral plating alone provides insufficient mechanical fracture stabilization. We intend to pursue future research in this endeavor to propose the best fixation method for our novel fracture model.
